# The Ultimaster Biodegradable-Polymer Sirolimus-Eluting Stent: An Updated Review of Clinical Evidence

**DOI:** 10.3390/ijms17091490

**Published:** 2016-09-06

**Authors:** Alberto Chisari, Anna Maria Pistritto, Raffaele Piccolo, Alessio La Manna, Gian Battista Danzi

**Affiliations:** 1 Division of Cardiology, Santa Corona Hospital, Via XXV Aprile, 38, Pietra Ligure 17027, Italy; albertochisari@yahoo.it; 2 Division of Cardiology, San Paolo Hospital, Savona 17100, Italy; annamariapistritto@hotmail.it; 3 Department of Cardiology, Bern University Hospital, University of Bern, Bern 3010, Switzerland; Raffaele.Piccolo@insel.ch; 4 Cardiovascular Department, Ferrarotto Hospital, University of Catania, Catania 95124, Italy; lamanna.cardio@gmail.com

**Keywords:** Ultimaster stent, biodegradable polymer drug eluting stent, new-generation DES, sirolimus-eluting stent, coronary stent

## Abstract

The Ultimaster coronary stent system (Terumo Corporation, Tokyo, Japan) represents a new iteration in drug-eluting stent (DES) technology that has recently received the Conformité Européenne (CE) mark approval for clinical use. The Ultimaster is a thin-strut, cobalt chromium, biodegradable-polymer, sirolimus-eluting coronary stent. The high elasticity of the biodegradable-polymer (PDLLA-PCL) and the abluminal gradient coating technology are additional novel features of this coronary device. The Ultimaster DES has undergone extensive clinical evaluation in two studies: The CENTURY I and II trials. Results from these two landmark studies suggested an excellent efficacy and safety profile of the Ultimaster DES across several lesion and patient subsets, with similar clinical outcomes to contemporary, new-generation DES. The aim of this review is to summarize the rationale behind this novel DES technology and to provide an update of available evidence about the clinical performance of the Ultimaster DES.

## 1. Introduction

Drug-eluting stents (DES) marked a breakthrough technology in the field of percutaneous coronary intervention (PCI) due to a profound reduction in neointimal hyperplasia and the need for repeat revascularization compared with bare-metal stents (BMS) [1,2,3]. Despite the initial enthusiasm, several concerns related to a higher risk of late thrombotic events and catch-up in efficacy during long-term follow-up hampered their widespread adoption in clinical practice [4,5,6,7,8,9,10,11,12,13,14].

Indeed, histopathological studies revealed a frequent occurrence of chronic inflammatory response to the early-generation of DES, particularly to the components of permanent polymers, which were found to delay vessel healing and stent endothelialization. As such, new-generation DES were developed with the specific aim to hone the safety profile of earlier devices. Newer generation DES were made with more biocompatible durable polymers or biodegradable-polymers (BP), which eliminated in turn the persistent inflammatory stimulus from the stent surface. Available evidence indicates that new-generation DES, including BP-DES, are safer and more effective than both BMS and early-generation DES, resulting in a paradigm shift in clinical practice [15,16,17,18,19,20]. As a result, new-generation DES are currently indicated in almost all lesion and patient subsets [21].

Notably, refinements in DES technology involved not solely the polymer, but also the stent platform and the choice of the antiproliferative agent. The availability of cobalt-chromium and platinum-chromium platforms remarkably decreased strut thickness, without affecting radial force and stent visibility. Since strut thickness is intertwined with neointimal hyperplasia and thrombogenicity [22,23], reducing stent thickness is key at improving the efficacy and safety profile of any coronary device. Finally, sirolimus and other analogues of the “limus family” have become the antiproliferative drugs of choice in new-generation DES, owing to greater efficacy than paclitaxel.

In this context, the Ultimaster coronary stent (Terumo Corporation, Tokyo, Japan), a new-generation, cobalt-chromium, biodegradable-polymer, sirolimus-eluting stent (BP-SES) has recently received the Conformité Européenne (CE) mark approval for clinical use. Therefore, the objective of this review is to illustrate this new stent technology and to summarize available data on the safety and efficacy of the Ultimaster BP-SES in patients undergoing PCI.

## 2. Stent Characteristics

The Ultimaster coronary stent consists of a cobalt-chromium platform coated with sirolimus (3.9 µg/mm stent length) in a matrix with bioresorbable, poly (dl-lactide-co-caprolactone) polymer (PDLLA/PCL = 90/10). Stent platform features a thin strut (80 µm), an open-cell 2-link design and uniform architecture for optimal coverage of bifurcation anatomy, which should facilitate the access to side branches and conformability to the vessel wall, while preserving a good radial force and radiopacity.

**Figure 1:**
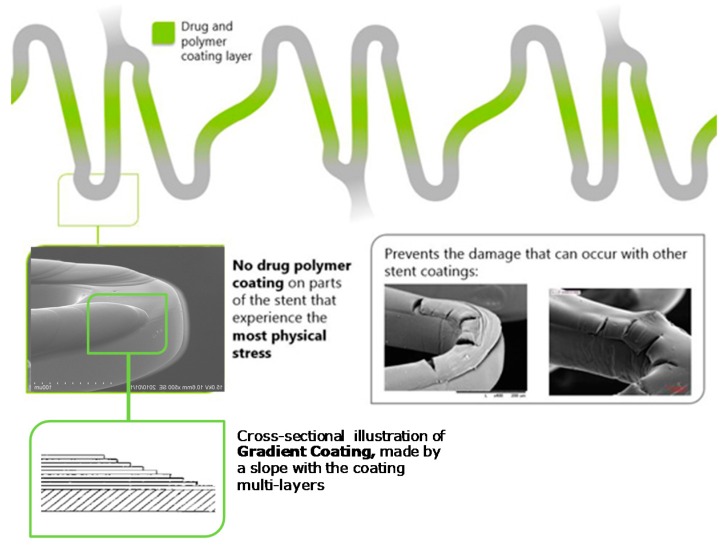
Gradient coating of Ultimaster coronary stent: Gradient coating consists in the absence of drug polymer on the hinges of the stent in order to reduce the risk of polymer damage that can occur with circumferential coating. The gradient coating is achieved by the multi-layer coating process, protected by Terumo’s patents.

A thin biocompatible, biodegradable gradient coating represents a novel feature in the stent design. Gradient coating consists of a lack of drug polymer on the stent areas experiencing the highest physical stress and was developed with the aim to reduce the risk of polymer cracking and delamination (Figure 1, on file at Terumo). The drug coating components were chosen to optimize the performance with minimal drug and polymer content and controlled drug release kinetics. The biodegradable PDLLA/PCL coating is metabolized through dl-lactide and caprolactone into carbon dioxide and water, and is expected to fully eliminate over three to four months.

**Figure 2:**
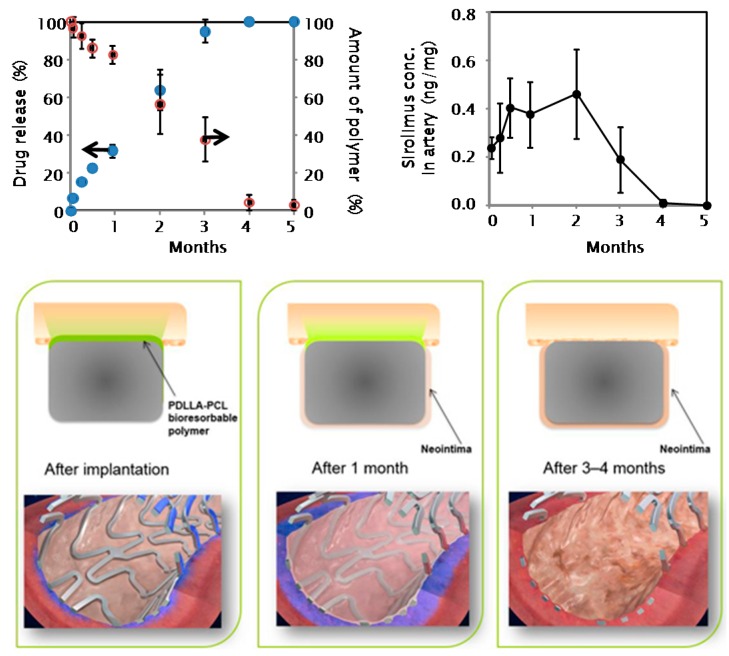
Process of polymer degradation after stent implantation: Bioresorbable polymer is metabolized into carbon dioxide and water and excreted via urine and feces within 3–4 months. Sirolimus is released along with polymer bioabsorption. After that time the Ultimaster drug-eluting stent is expected to behave as a thin strut bare metal stent. Blue spots indicate % drug release over time; red circles indicate % amount of polymer remaining on stent surface.

The presence of abluminal instead of circumferential coating confers the dual advantage of (a) reducing the overall drug load in view of a targeted abluminal drug delivery; and (b) enhancing strut endothelialization by leaving the luminal side of the stent free from drug and polymer. The initial drug burst is needed to suppress vascular injury and inflammation induced by the catheter manipulation and stent implantation, while the remaining drug is released simultaneously to polymer biodegradation for three months. Thereafter, the Ultimaster BP-SES is expected to become a bare metal stent because only the metal backbone remains in situ (Figure 2, on file at Terumo).

The stent is mounted on a rapid-exchange catheter with a high-pressure, semi-compliant balloon. The Ultimaster BP-SES is available in diameters of 2.25, 2.50, 2.75, 3.00, 3.50 and 4.00 mm, and in length sizes of 9, 12, 15, 18, 24, 28, 33 and 38 mm.

## 3. Clinical Trials

### 3.1. CENTURY Study

The CENTURY (Clinical Evaluation of New Terumo Drug-Eluting Coronary Stent System in the Treatment of Patients with Coronary Artery Disease) was a multicenter, single-arm, prospective study that enrolled 105 patients (113 lesions) with stable coronary artery disease [24]. Complex coronary lesions represented a key exclusion criterion.

All patients were scheduled to undergo follow-up angiography at six months. In 40% and 20% of patients, intravascular ultrasound (IVUS) and optical coherence tomography (OCT) were performed as well. The primary endpoint was in-stent late lumen loss at six-month follow-up angiography. Secondary endpoints included clinical, IVUS and OCT outcomes. All secondary angiographic, IVUS and OCT endpoints were assessed at six months. Secondary clinical endpoints were assessed at 1, 6, 12 and 24 months and yearly up to five years and included target lesion failure (TLF), defined as the composite of cardiac death, target-vessel myocardial infarction (MI), or clinically-indicated target-lesion revascularization (TLR). Each component of TLF and stent thrombosis (ST), according to Academic Research Consortium (ARC) criteria, was additionally evaluated.

At six months, angiographic late lumen loss was 0.04 mm ± 0.35 mm and the rate of binary restenosis amounted to 0.9%. Consistently, IVUS-assessed neointimal volume obstruction was 1.02% ± 1.62%. Furthermore, IVUS analysis showed the absence of vessel remodeling with a similar peri-stent vessel volume after stent implantation (313.10 mm^3^ ± 128.30 mm^3^) and at six-month follow-up (312.67 mm^3^ ± 142.44 mm^3^). At OCT assessment, strut coverage was complete in 96.2% of analyzed struts, with 1.66 ± 4.02 malapposed stent struts.

The one-year rate of TLF and TLR was 3.8% and 1.9%, respectively. At two-year follow-up, TLF and TLR occurred in 5.7% and 2.8% of patients. There was one acute definite ST and no cases of late or very late ST up to two-year follow-up.

### 3.2. CENTURY II Trial

The CENTURY II was a prospective, multicenter, randomized, single-blind, non-inferiority trial conducted in Europe, Korea, and Japan [25]. Study eligibility consisted of ischemic heart disease due to coronary lesions amenable for treatment with stents ≥2.5 and ≤4.0 mm (≤3.5 mm in Japan). Patients were randomly in a 1:1 ratio to receive the Ultimaster BP-SES or permanent-polymer everolimus-eluting stent (Xience EES, Abbott Vascular, Santa Clara, CA, USA). The analysis was conducted on two cohorts of patients to comply with Japanese regulations: the total population (TP) and the Japanese Requirement population (Cohort JR).

The primary endpoint of the trial was TLF, a device-oriented composite endpoint including cardiac death, any target-vessel MI, or clinically-indicated TLR, at nine months follow-up. Secondary endpoints included: (a) target-vessel failure (TVF), a composite of cardiac death, any target-vessel MI, or clinically-indicated target-vessel revascularization (TVR); (b) a patient-oriented composite endpoint including all-cause death, any MI, or any coronary revascularization; (c) the composite of cardiac death or MI; (d) each single component of the device- and patient-oriented composite endpoints; and (e) bleeding according to Bleeding Academic Research Consortium criteria.

Primary angiographic endpoints were in-stent and in-segment binary restenosis (≥50% diameter stenosis), as well as in-stent and in-segment late lumen loss at nine months after PCI. Angiography at nine-month follow-up was scheduled for 400 participants of the Japanese Cohort.

Overall, a total of 1119 patients undergoing PCI were included in the intention-to-treat analysis (562 patients randomized to the Ultimaster BP-SES and 557 patients randomized to EES). In the per-protocol analysis, 1101 patients were analyzed (551 allocated to Ultimaster BP-SES and 550 allocated EES). Baseline clinical characteristics, angiographic features and lesion complexity were similar in both cohorts and in both study arms. Consistent with the eligibility criteria, high-risk acute coronary syndrome was less frequently an indication to PCI for the Japanese cohort, in whom acute MI was an exclusion criterion (along with left main disease and bypass graft stenosis). More than 80% of the lesions were classified as B2 or C, 14% were located at bifurcation, 7% were ostial, and 5% had 100% occlusion. Multivessel PCI was performed in 16% of patients, whereas multiple lesions were treated in 25% of cases. Except for longer lesions in the Utimaster BP-SES arm, angiographic and procedural characteristics were well balanced.

The CENTURY II trial proved the non-inferiority of the Ultimaster BP-SES to the Xience EES. In the overall cohort, 95.6% of patients randomized to the Ultimaster BP-SES and 95.1% of patients randomized to EES were free from TLF at nine-month follow-up. Main results established the non-inferiority of the Ultimaster BP-SES, with an absolute risk difference of 0.55% favoring the experimental group (*p* < 0.0001 for non-inferiority). In the Cohort JR, the freedom from TLF was 95.9% in patients randomized to the Ultimaster BP-SES and 94.6% in patients randomized to EES, respectively, and non-inferiority was also confirmed because of an absolute difference of 1.24% in favor of BP-SES (*p* for non-inferiority <0.0005). The per-protocol analysis yielded consistent results. Moreover, there was no significant difference between the Ultimaster BP-SES and EES with respect to secondary endpoints. The rate of TLR “per lesion” up to nine months was 1.7% (12/711) in the Ultimaster BP-SES group and 2.1% (15/716) in the EES group (*p* = 0.56).

In the overall cohort, ST throughout nine months was observed in 10 patients (five cases for each group). All ST events were definite ST yielding a rate of 0.9% per group (*p* = 0.99). In-stent late lumen loss was 0.26 mm in the Ultimaster BP-SES group compared with 0.18 mm in the EES group (*p* = 0.003). At variance, in-segment late lumen loss (0.09 mm vs. 0.10 mm) and binary restenosis (1.21% vs. 1.27% in-stent restenosis; 2.83% vs. 3.80% in-segment restenosis) were not significantly different between the Ultimaster BP-SES and EES.

Overall, the Ultimaster BP-SES was found as safe and effective as the Xience EES in in nearly “all comers” patients undergoing contemporary PCI.

## 4. Subgroup Analyses

Pre-specified analyses of the CENTURY II trial included the following subsets: Bifurcation lesions, small vessel disease, long lesions, and acute coronary syndromes.

### 4.1. Bifurcation Lesions

A total of 194/1119 patients (17.3%) underwent PCI of bifurcation lesion (95 patients with BP-SES vs. 99 patients with EES) [26]. Overall, baseline clinical characteristics, clinical presentation and angiographic characteristics were similar in both study arms. The majority of bifurcation lesions (74.5%) were classified as “true” bifurcations. The Medina bifurcation categories were well distributed, though Medina 1,0,1 was more frequent in the EES group (*p* = 0.04). A single stent technique was the most common PCI technique and was equally used in both groups. Device and procedure success amounted to 99.3% and 97.9%, respectively. Freedom from TLR at one year occurred in 94.7% (90/95) patients in the Ultimaster BP-SES and 91.9% (91/99) in the PP-EES groups, supporting the non-inferiority of the BP-SES with respect to EES in this lesion subset (*p* for non-inferiority = 0.031). The one-year rate of clinically-driven TLR was 3.2% for BP-SES and 3% for EES (*p* = 0.96) and clinically-driven TVR was 4.2% for BP-SES and 4% for EES (*p* = 0.95). No significant differences were observed with respect to secondary endpoints. The one-year rate of TVF was 6.3% in the BP-SES and 9.1% in the EES group (*p* = 0.47). The patient-oriented composite endpoint occurred in 9.5% patients of the BP-SES arm versus 18.2% patients of the EES arm (*p* = 0.08). During one-year follow-up, definite or probable ST occurred in three patients, 1/95 (1.1%) in the BP-SES arm, and 2/99 (2%) in the EES arm (*p* = 0.58).

In conclusion, this substudy showed favorable outcomes, with a low rate of TLF and ST among patients undergoing PCI in coronary bifurcation.

### 4.2. Small Vessel Disease

The CENTURY II study included 525 patients with a reference vessel diameter ≤2.5 mm. A total of 277 patients were treated with BP-SES and 248 with EES [27]. There was no significant difference between treatment groups in baseline or procedural data. Mean pre-procedural reference diameter (2.30 ± 0.40 vs. 2.31 mm ± 0.42 mm, BP-SES vs. EES, *p* = 0.59) and stented length (24.0 ± 11.7 vs. 23.5 mm ± 11.5 mm, BP-SES vs. EES, *p* = 0.45) were similar between the two groups. At 12 months, there was no significant difference between the Ultimaster BP-SES and EES groups with respect to TLF (6.9% vs. 7.7%, *p* = 0.72), cardiac death (1.1% vs. 1.2%, *p* = 0.90), target-vessel MI (1.8% vs. 3.2%, *p* = 0.30), TLR (4.0% vs. 5.7%, *p* = 0.37), and definite or probable ST (0.7% vs. 1.2%, *p* = 0.57).

Taken together, these findings indicate that the Utimaster BP-SES showed a comparable safety and efficacy profile to the EES for the treatment of small vessel coronary artery disease.

### 4.3. Long Lesions

A total of 182/1119 patients (16.2%) had at least one long coronary lesion, defined as lesion length ≥25 mm [28]. Of them, 101 were treated with the Ultimaster BP-SES and 81 with EES. Baseline patient and lesion characteristics were well balanced between groups. There was no difference between BP-SES and EES in lesion length (29.4 ± 12.5 vs. 29.1 mm ± 11.4 mm, *p* = 0.76), number of treated lesions per patient (1.58 ± 0.78 vs. 1.53 ± 0.76, *p* = 0.65), and number of stents implanted per lesion (1.5 ± 0.6 vs. 1.6 ± 0.7, *p* = 0.23). Device and procedural success were high for both groups (BP-SES vs. PP-EES: 100% vs. 99.2% and 97.0% vs. 97.5%). At nine months, cardiac death (2.0% vs. 1.2%, *p* = 0.70), MI (3.0% vs. 4.9%, *p* = 0.49), clinically-driven TLR (2.0% vs. 3.7%, *p* = 0.48) and TLF (6.9% vs. 8.6%, *p* = 0.67) occurred at similar rate among patients randomized to the Ultimaster BP-SES versus EES. There was trend toward a lower risk of any TLR showed favoring the Ultimaster BP-SES (3.0% vs. 9.9%, *p* = 0.053). There was only one case (1.2%) of subacute ST, occurring in the EES group.

Therefore, the overall safety and efficacy profile of the Ultimaster BP-SES was similar to the EES among patients undergoing PCI of long coronary lesions.

### 4.4. Acute Coronary Syndromes (ACS)

Out of 1119 patients enrolled in CENTURY II, 264 were high-risk ACS patients, which were randomly assigned to receive BP-SES (*n* = 126) or EES (*n* = 138) [29]. In this subgroup, 23.1% were patients presented with ST-segment elevation MI, whereas 76.9% with Non-ST-segment elevation MI. Baseline clinical, angiographic and procedural characteristics were similar between the two groups. TLF occurred in 6.3% (8/126) of patients receiving BP-SES and 6.5% (9/138) of patients receiving EES (*p* = 0.95). Individual components of TLF showed comparable rates of cardiac death (0.0% in BP-SES and 2.1% in EES, *p* = 0.10), target-vessel MI (0.7% in BP-SES and 3.6% in EES, *p* = 0.12), and clinically-driven TLR (4.8% BP-SES and 3.6% EES, *p* = 0.64) at 24-month follow-up. TVF occurred in 6.3% (8/126) of patients receiving BP-SES and 9.4% (13/138) of patients receiving EES (*p* = 0.36). ST occurred in two patients (1.59%) treated with BP-SES, and in one patient (0.72%) treated with EES (*p* = 0.51); all events were subacute. Such findings confirm that the use of the Ultimaster BP-SES is associated with similar outcomes as compared with Xience EES among patients presenting with high-risk ACS.

### 4.5. OCT Substudy

This study compared tissue coverage in coronary lesions treated with the Ultimaster BP-SES and Xience EES [30]. Twenty-seven patients (13 BP-SES and 14 EES) undergoing OCT at nine-month follow-up were included. Baseline patient and lesion characteristics were similar between the two groups. A total of 6450 struts were analyzed (BP-SES, *n* = 2951; EES, *n* = 3499). Mean neointimal thickness did not significantly differ between both groups (110 ± 10 in BP-SES vs. 93 ± 9 µm in PP-EES; *p* = 0.22). No significant differences in neointimal volume obstruction (13.2% ± 4.6% in BP-SES vs. 10.5% ± 4.9% in PP-EES, *p* = 0.14) or other areas-volumetric parameters were noted. Thirty and 79 uncovered struts (1.02% and 2.26%; *p* = 0.35), and three and four malapposed struts (0.10% and 0.11%; *p* = 0.94) were reported in the BP-SES and EES groups, respectively. At nine-month follow-up, the OCT sub-study of the CENTURY II trial suggested a remarkable tissue coverage and apposition of the Ultimaster BP-SES.

These findings support that the Ultimaster BP-SES has an excellent vascular healing response akin to EES.

## 5. Discussion

The novel Ultimaster coronary stent is a new-generation, thin-strut (80 µm), cobalt-chromium, BP-SES. The drug is released for three months, leaving a thin-strut backbone inside the vessel. The availability of a device that combines the advantage of new-generation DES, in terms of reduction of restenosis and TLR, with the long-term safety of BMS, regarding the risk of late ST, is highly attractive. It is well known that chronic inflammation to components of the permanent polymer represents an important factor associated with an increased risk of late DES failure and ST. Hypersensitivity reactions to the polymers may lead to delayed vessel healing and stent coverage by non-functional endothelium, factors implied in the main drawbacks of first-generation DES, such as stent thrombosis, restenotic late catch-up and vessel remodeling [10,11,31,32,33,34,35]. To overcome these limitations, biocompatible and biodegradable polymers have been developed in the context of newer generation DES. Nevertheless, at this point in time, it remains unclear as to whether BP-DES really improves long-term clinical outcomes in comparison to permanent polymer, new-generation counterparts. Indeed, a network meta-analysis of randomized trials showed that biodegradable polymer biolimus-eluting stent may even increase the risk of definite or probable ST as compared with EES [36]. As a result, a preferential use of biodegradable polymer DES cannot be yet recommended. Arguably, the safety and efficacy profile of a coronary device is influenced not solely by the type of polymer, but also by a variety of other factors (stent alloy, design, strut thickness, and drug). In contrast to the biolimus-eluting stent, the Ultimaster BP-SES features a different stent alloy (cobalt chromium vs. stainless steel), a thinner strut thickness (80 vs. 120 μm), faster biodegradable polymer (3–4 vs. 9–12 months) and analogue drug (sirolimus vs. biolimus) [37,38]. Such characteristics are expected to translate into better clinical outcomes. The Ultimaster design hypothesis is convincingly supported by clinical evidence from a comprehensive research program [39]. The results of the CENTURY study confirmed high efficacy of the Ultimaster BP-SES with a late loss of 0.04 mm at six months. This late loss is in line with the late loss reported for contemporary DES using limus drugs, such as the Xience EES (0.12 mm), the Orsiro BP-SES (0.05 mm, Biotronik AG, Bülach, Switzerland), the SYNERGY BP-EES (0.10 mm, Boston Scientific, Natick, MA, USA) and the PROMUS Element EES (0.15 mm, Boston Scientific). The clinical benefit of the Ultimaster BP-SES was reflected in low rates of clinically indicated revascularizations of target lesions up to two years. The rate of TLF at one and two years (3.8% and 5.7%, respectively) was in line with other contemporary drug-eluting stents. IVUS and OCT findings lend support to the favorable efficacy and safety profile of Ultimaster BP-SES by revealing a very thin and homogenous layer of neointima covering the stent struts, resulting in an average strut coverage rate of 96%. Furthermore, no signs of vessel enlargement, ectasia or aneurysms were observed at follow-up. As such, no significant inflammatory reactions of the vessel wall occur after Ultimaster BP-SES implantation [24]. 

The CENTURY II trial is a global, multicenter, randomized study that compared the Ultimaster BP-SES against the Xience EES among 1123 patients with broad inclusion criteria. At nine months the Ultimaster BP-SES was non-inferior to the Xience EES. Mid-term safety was well supported in view of similar rates of cardiac death, MI, and ST between the Ultimaster BP-SES and EES, along with a rare ST occurrence [24]. Moreover, the analysis of heterogeneity corroborated the main study findings across many patient and lesion subsets [26,27,28,29].

Potential benefits related to the use of a biodegradable polymer DES should theoretically appear over long-term follow-up. Indeed, the five-year follow-up of LEADERS trial showed that the incidence of ST associated with the BP-BES was almost plateaued after one year, whilst ST continued to occurred beyond one year among patients treated with the durable-polymer SES [40]. However, whether the use of a biodegradable instead of durable polymer will result in improved late outcomes has to be yet determined. Therefore, further long-term follow-up is required to evaluate the long-term safety and efficacy of the Ultimaster BP-SES.

## 6. Conclusions

The new, thin strut, biodegradable polymer, sirolimus-eluting stent resulted to be as safe and as effective as everolimus-eluting stent in a complex patient population. Ongoing studies have been designed to confirm the excellent performance of the Ultimaster DES among patients with acute myocardial infarction (MASTER study, NCT02828683), and to assess potential for reduced dual antiplatelet therapy (DISCOVERY 1TO3, NCT01844843) or to investigate the performance among consecutive patients undergoing PCI in daily clinical practice (e-ULTIMASTER, NCT02188355).

Results from these ongoing studies, as well as from the long-term follow-up of the conducted trials, are expected to provide new insights in the clinical application of the Ultimaster BP-SES in the era of new-generation DES.
